# Chronic viral infections in myalgic encephalomyelitis/chronic fatigue syndrome (ME/CFS)

**DOI:** 10.1186/s12967-018-1644-y

**Published:** 2018-10-01

**Authors:** Santa Rasa, Zaiga Nora-Krukle, Nina Henning, Eva Eliassen, Evelina Shikova, Thomas Harrer, Carmen Scheibenbogen, Modra Murovska, Bhupesh K. Prusty, the European Network on ME/CFS (EUROMENE)

**Affiliations:** 1 Institute of Microbiology and Virology, Rīga Stradiņš University, Riga, Latvia; 2 Biocenter, Chair of Microbiology, University of Würzburg, Würzburg, Germany; 3 Institute for Virology and Immunobiology, Würzburg, Germany; 4 Department of Virology, National Center of Infectious and Parasitic Diseases, Sofia, Bulgaria; 5 Department of Internal Medicine 3, Universitätsklinikum Erlangen, Friedrich-Alexander-University Erlangen-Nürnberg (FAU), Erlangen, Germany; 6 Institute for Medical Immunology, Charité-Universitätsmedizin Berlin, Campus Virchow, Berlin, Germany

**Keywords:** ME/CFS, Viral infections, Biomarkers

## Abstract

**Background and main text:**

Myalgic encephalomyelitis/chronic fatigue syndrome (ME/CFS) is a complex and controversial clinical condition without having established causative factors. Increasing numbers of cases during past decade have created awareness among patients as well as healthcare professionals. Chronic viral infection as a cause of ME/CFS has long been debated. However, lack of large studies involving well-designed patient groups and validated experimental set ups have hindered our knowledge about this disease. Moreover, recent developments regarding molecular mechanism of pathogenesis of various infectious agents cast doubts over validity of several of the past studies.

**Conclusions:**

This review aims to compile all the studies done so far to investigate various viral agents that could be associated with ME/CFS. Furthermore, we suggest strategies to better design future studies on the role of viral infections in ME/CFS.

## Background

Myalgic encephalomyelitis/chronic fatigue syndrome (ME/CFS) is a disease that causes central nervous system (CNS) and immune system disturbances, cell energy metabolisms and ion transport dysfunction, as well as cardiovascular problems, gastrointestinal dysfunction, cognitive impairment, myalgia, arthralgia, orthostatic intolerance, inflammation and innate immunity disturbances. The main clinical sign is persisting chronic fatigue, which is not relieved by rest and lasts for more than 6 months [1]. A large group of patients remains wheelchair-dependent and many remain housebound or even bedbound [2].

ME/CFS is sporadic with occasional outbreaks [3]. Currently around 80% of ME/CFS cases are undiagnosed [4]. According to the available literature, already back in 2009 around 17 million people were diagnosed with this disease, including 800,000 patients in the United States of America and 240,000 in the United Kingdom [5]. Etiological factors for ME/CFS include genetic predisposition, stress, trauma, exposure to toxins, physical activity and rest ratio, as well as a recent history of infectious disease [2]. Females within the age group of 30–39 years are more prone to this disease [6]. Nevertheless, ME/CFS can affect individuals from all races, genders, age groups and social statuses. Population studies show that the prevalence of ME/CFS worldwide is from 0.2% of clinically diagnosed up to 3.48% of self-reported population depending on the applied diagnostic criteria. Most of the patients with ME/CFS suffer from long lasting symptoms, with only 6% of patients experiencing remission of the disease [7–9]. ME/CFS symptoms range from long lasting fatigue, memory loss, difficulty concentrating, sore throat, lymphadenopathy, muscle pain, headaches and un-refreshing sleep to extreme fatigue after exertion. The pathomechanisms of ME/CFS are still unknown, and there are no standardized biological markers or tests for diagnostics; therefore, even the existence of this medical diagnosis has been questioned for long time [10].

During the past 2 decades, few illnesses have been as extensively discussed as chronic fatigue syndrome (CFS). A consensus for the proper diagnostic definition for CFS was reached in 1994 on a case definition from the United States Centers for Disease Control and Prevention [11]. Therefore, prolonged fatigue is defined as self-reported, persistent fatigue lasting 1 month whereas chronic fatigue is persistent or relapsing fatigue lasting 6 months or longer [11]. Later in 2011 International Consensus Panel (ICP) developed International Consensus Criteria suggesting that this disease is to be defined as myalgic encephalomyelitis (ME) due to neuropathological inflammation [12].

Clinical evaluation for identifying underlying or contributing conditions of chronic fatigue is required before further diagnosis or classifications can be made. Besides persistent post-exertional fatigue, ME/CFS is characterized by substantial symptoms related to cognitive, immune and autonomous dysfunctions [13, 14]. Though ME/CFS is a chronic illness of uncertain cause with unknown pathogenesis, there is abundant evidence of an underlying biological process. Since sudden disease outbreaks in patients start with a “flu-like” illness, it seems plausible that an infectious agent can trigger the syndrome. In fact, viral-like illnesses appear to precede ME/CFS onset in approximately 50% of patients [15]. In addition, there are indications that immunological dysfunction may contribute to the emergence of symptoms [16].

The pathogenesis of ME/CFS is likely multi-factorial and various microbial and viral infections are considered to be the possible trigger factors of ME/CFS. The illness has been frequently accompanied with various viral infections and studies have been conducted on association of ME/CFS with Epstein–Barr virus (EBV) [17, 18], cytomegalovirus (CMV) [19], human herpesvirus (HHV) 6, HHV-7, HHV-8 [20–22], human parvovirus B19 (B19V), enteroviruses [23], lentivirus [24] and bacteria as mycoplasma [25], Lyme disease causing borrelia, Q fever causing *Coxiella burnetii* [26] and other pathogens.

Still, the association of ME/CFS with a single infectious agent has not been confirmed, and the role of viral infections in ME/CFS remains obscure [2, 27]. This may be attributed to the small size and/or heterogeneity of studied ME/CFS populations, not well-characterized ME/CFS patients, lack of adequate controls, high prevalence of persistent viral infection in the general population, different methodological approaches applied and so on. Here we present an overview of studies addressing the possible association of viral infections with ME/CFS, focusing on human herpesviruses, B19V, and enteroviruses, which all can trigger ME/CFS. These viruses, after an acute infection, remain in the body as mostly latent, persistent infections and may reactivate under various conditions. Immunologic disturbance associated with ME/CFS may be the result of viral infection or may lead to reactivation of latent viruses. Once reactivated, the viruses may contribute to the morbidity of ME/CFS via inflammation and immune dysregulation, especially the herpesviruses EBV and HHV-6, which infect immune cells [28]. Viral infections can trigger an autoimmune response as well [29]. In the majority of ME/CFS cases, there is no conclusive evidence for chronic viral infection, but it is plausible that viruses could act via a “hit and run” mechanism; this theory proposes that viruses trigger the disease, cause immune abnormalities and leave a dysfunctional immune system and/or autoimmunity.

## Human herpesviruses

According to the International Committee on Taxonomy of Viruses, *Herpesviridae* family includes *Alphaherpesvirinae*, *Betaherpesvirinae* and *Gammaherpesvirinae* subfamilies. Currently there are nine human herpesviruses: herpes simplex virus (HSV)-1, HSV-2, varicella zoster virus (VZV), EBV, CMV, HHV-6A, HHV-6B, HHV-7, HHV-8. HHV-6 and HHV-7, members of the *Herpesviridae* family*, Beta*-*herpesvirinae* subfamily*, Roseolovirus* genus, are most studied human pathogens in association with ME/CFS [30]. HHV-6A was first isolated in 1986 from peripheral blood mononuclear cells (PBMCs) of patients with acquired immunodeficiency syndrome (AIDS) and lympholeukosis [31]. HHV-7 was first isolated in 1990 from CD4^+^ lymphocytes of healthy adult [32]. Primary infection with these viruses usually is observed in early childhood—from age of 6 months to 3 years. They can cause *Roseola infantum* or *Exanthema subitum* with fever, rashes and elevated body temperature lasting for 3 to 5 days. In addition, it can affect several organ systems, including CNS [33]. Likewise, infection course can be asymptomatic [34]. HHV-6B and HHV-7 are widespread and prevalence is more than 90% of general population [35]. The seroprevalence of HHV-6A is unknown. However one study of Chinese individuals put the prevalence at 11% of controls [36]. One feature of all herpesviruses is that they can establish a lifelong persistent infection termed latency [37]. Latent HHV-6 can reside in a dormant state inside human host cells by integrating viral genome into telomeric ends of host cell chromosomes (ciHHV-6) [38]. Recently also chromosomal integration of HHV-7 into the host genome was discovered [39]. During latency, minimal viral transcription and no production of infectious virions occurs, resulting in no detectable clinical complications. However, activated forms of HHV-6A/B and HHV-7 are known to have immunomodulating properties such as modulating the expression of several cytokines and chemokines or inducing immunosuppression by triggering apoptosis in lymphocytes [40–42]. Since both viruses are ubiquitous, concurrent infection is common and it has also been reported, that HHV-7 can reactivate HHV-6A/B [43–45].

Reactivation of latent HHV-6A/B can be caused by various stress conditions leading to increased severities of multiple human disorders [46, 47]. Even though evidence is lacking, it is also possible that exposure to certain drugs could reactive ciHHV-6. One known chemical to reactivate HHV-6 in vitro is the HDAC inhibitor Trichostatin A [48]. Similarly two other commonly known pharmaceuticals, sodium valporate and amoxicillin, also enhance HHV-6 replication [49, 50]. Virus reactivation is possible in cases of immune disturbances, long-term stress, immunosuppressive therapy, prolonged anaesthesia, transplantation, AIDS and others [21]. For example, HHV-6A reactivation has been found in patients with multiple sclerosis (MS) [51] and HIV infection [52] and HHV-6B reactivation has occurred in amnesia [53] and hepatitis [54]. The virus can also reactivate in the presence of malignant and non-malignant diseases [55] and HHV-6A antibodies are a risk factor for non-Hodgkin lymphoma [36].

Due to its life-long persistence and its broad tissue tropism, HHV-6 has been speculated to be a possible trigger for ME/CFS. The involvement of herpesviruses in ME/CFS was already considered in 1988, when serological evidence pointed to reactivation of HHV-6 among patients, and a subset of patients were found to exhibit higher levels of antibodies against the EBV viral capsid antigen (VCA) and early antigen (EA) but an absence of antibodies to EBNA [56]. Based on earlier reports and presentations, Ablashi suggested in 1994 that ME/CFS could be a result of immunological disturbances after herpesvirus reactivation [57]. Investigating this hypothesis, Buchwald et al. started the first large study [20], when the case definition for ME/CFS had not yet been made. Their study included 259 patients with ME/CFS symptoms. Serum chemistry tests and polymerase chain reaction (PCR) assays confirmed the finding of active HHV-6 infection in 70% of patients. Even though they suggested that active replication of HHV-6 represented reactivation of latent infection due to immunologic dysfunction, they did not directly address HHV-6 playing a role in producing ME/CFS symptoms.

Follow-up studies using only serological techniques could not differentiate between active and latent infection [21, 22] but revealed a slight tendency to an association between ME/CFS and HHV-6, with a greater prevalence and higher levels of HHV-6-specific antibodies in patients, as well as a greater prevalence of DNA in PBMCs from those with ME/CFS. Notably, in both of these studies, HHV-6A was found to predominate among ME/CFS patients. Moreover, an association between active HHV-6 infection and ME/CFS has been demonstrated in studies distinguishing between active and latent infection using immunofluorescence assays directed against HHV-6A antigens or early antibody assays [58, 59]. On the other hand, there are several studies in which both serological techniques as well as PCR-based techniques distinguishing between active and latent infection lead to the conclusion that HHV-6 infection does not correlate with ME/CFS [60, 61]. Chapenko et al. [62] also evaluated whether HHV-6 infections could be the causative agent for ME/CFS. They found active HHV-6 more often in ME/CFS patients than controls, and active infection correlated with the occurrence of the clinical symptoms of ME/CFS, including lymphadenopathy, subfebrility, and malaise after exertion.

The presence of HHV-7 and other HHVs has been determined in patients with ME/CFS. Sairenji et al. revealed 100% HHV-7 seropositivity among 20 patients [63]. In another study involving 17 ME/CFS patients, HHV-7 reactivation was detected more frequently than HHV-6 reactivation and concurrent active HHV-6 and HHV-7 infection was accompanied by immunological changes in the form of significantly increased CD95^+^ cells, decreased CD3^+^ and CD4^+^ T cells, and a lower CD4/CD8 ratio [64]. Active HHV-6, HHV-7 and B19V infection/co-infection was confirmed analysing 108 patients with ME/CFS [62].

However, PCR analysis showed a similarly high detection rate of HHV-7 genomic sequences among patients with ME/CFS and controls (82% vs 83%) [65]. In another study, the percentage of HHV-6, HHV-7 and co-infection was similar between patients and controls; nevertheless, HHV-7 was approximately two times more prevalent than HHV-6, with HHV-6 and HHV-7 DNA detected in the PBMCs of 26.7% and 69.9% of healthy adults, respectively, and 35.1% and 77.3% of ME/CFS patients [66]. Others found HHV-7 DNA in only 7.7% of peripheral blood lymphocyte samples from patients with ME/CFS using PCR [67]. In addition, detection of HHV-7 in a high percentage of gastro-intestinal biopsies from patients (85–92%) and controls (66–83%) with quantitative PCR (qPCR) is reported, though without statistical difference between the groups [68]. No difference between severity of symptoms and viral load of HHV-6 and HHV-7 in saliva and PBMC of ME/CFS patients and controls was shown [69]. Besides, detectable reactivation of HHV-6A/6B and HHV-7 in saliva is considered as a biomarker for physiological fatigue, and can therefore be used to distinguish between pathological and physiological fatigue [70].

There is no statistically significant difference between reported studies that have found no correlation between HHV-6/HHV-7 infection/co-infection and ME/CFS (in total 17) and publications that noted a correlation (in total 12) (P = 0.2935) (Table 1). Nevertheless, some study cohorts are rather small to draw general conclusions about the association of a viral infection with the disease.

**Table 1:** Publications on analysis of human herpesvirus 6 and/or 7 in myalgic encephalomyelitis/chronic fatigue syndrome

Publication title	Author	Year	Study participants	Sample type	Method	Correlation assumed
HHV-6 reactivation in chronic fatigue syndrome	Josephs et al.	1991	7 patients, 2 controls	PBMCs	IFA, Southern blot	Yes
A chronic illness characterized by fatigue, neurologic and immunologic disorders, and active human herpesvirus type 6 infection	Buchwald et al.	1992	259 patients, 47 controls	Serum, PBMCs	PCR, ELISA	Yes
Prevalence of human herpesvirus 6 variants A and B in patients with chronic fatigue syndrome	Yalcin et al.	1994	13 patients, 13 controls	Serum, PBMCs	PCR	Yes
Antibody responses to Epstein–Barr virus, human herpesvirus 6 and human herpesvirus 7 in patients with chronic fatigue syndrome	Sairenji et al.	1995	20 patients, 26 controls	Serum	IFA	Yes
Prevalence of IgM antibodies to human herpesvirus 6 early antigen (p41/38) in patients with chronic fatigue syndrome	Patnaik et al.	1995	154 patients, 165 controls	Serum	ELISA	Yes
Active HHV-6 infection in chronic fatigue syndrome patients from Italy: new data	Zorzenon et al.	1996	52 patients, 51 controls	Serum, PBMCs	IFA, PCR	Yes
Frequent HHV-6 reactivation in multiple sclerosis (MS) and chronic fatigue syndrome (CFS) patients	Ablashi et al.	2000	35 patients, 28 controls	Serum, PBMCs	ELISA, IFA	Yes
Dynamics of chronic active herpesvirus-6 infection in patients with chronic fatigue syndrome: data acquisition for computer modeling	Krueger et al.	2001	10 patients	Serum, blood	ELISA, qPCR	Yes
Activation of human herpesviruses 6 and 7 in patients with chronic fatigue syndrome	Chapenko et al.	2006	17 patients, 12 patients with UCF, 20 controls	Plasma, serum, PBMCs	nPCR, flow cytometry	Yes
Association of active human herpesvirus-6, -7 and Parvovirus B19 infection with clinical outcomes in patients with myalgic encephalomyelitis/chronic fatigue syndrome	Chapenko et al.	2012	108 patients, 90 controls	Plasma, serum	ELISA, nPCR, qPCR, REA	Yes
Myalgic encephalomyelitis/chronic fatigue syndrome and gulf war illness patients exhibit increased humoral responses to the herpesvirus-encoded dUTPase: implications in disease pathophysiology	Halpin et al.	2017	74 patients, 151 controls	Serum	ELISA	Yes
Human herpesvirus 6 and human herpesvirus 7 in chronic fatigue syndrome	Di Luca et al.	1995	36 patients, 24 controls	Plasma, PBMCs	PCR	No: HHV-7 Yes: HHV-6A
Chronic fatigue syndrome: clinical condition associated with immune activation	Landay et al.	1991	63 patients, 40 controls	Serum, plasma	IFA	No
A comprehensive immunological analysis in chronic fatigue syndrome	Gupta , Vayuvegula	1991	20 patients, 20 controls	PBMCs, serum	FACS, IFA	No
Clinical, epidemiologic, and virologic studies in four clusters of the chronic fatigue syndrome	Levine et al.	1992	31 patients, 105 controls	Serum	IFA, western blot, PCR	No
Simultaneous measurement of antibodies to Epstein–Barr virus, human herpesvirus 6, herpes simplex virus types 1 and 2, and 14 enteroviruses in chronic fatigue syndrome: is there evidence of activation of a nonspecific polyclonal immune response?	Manian	1994	20 patients, 20 controls	Serum	IFA	No
Detection of human herpesvirus 6 in plasma of children with primary infection and immunosuppressed patients by polymerase chain reaction	Secchiero et al.	1995	39 patients, 37 controls	Plasma or serum	PCR	No
Chronic fatigue syndrome (CFS): a critical evaluation of testing for active human herpesvirus-6 (HHV-6) infection	Wagner et al.	1996	107 patients	Serum, PBMCs	IFA, ELISA, nPCR	No
Viral serologies in patients with chronic fatigue and chronic fatigue syndrome	Buchwald et al.	1996	548 patients (CFS, CF and FM), 30 controls	Serum	ELISA, western blot, IFA	No
Human herpesviruses in chronic fatigue syndrome	Wallace	1999	76 patients, 73 controls	Serum	PCR	No
Human herpesvirus 6 and 7 in chronic fatigue syndrome: a case–control study	Reeves et al.	2000	26 patients, 50 controls	Serum	PCR	No
No evidence of active infection with human herpesvirus 6 (HHV-6) or HHV-8 in chronic fatigue syndrome	Enbom et al.	2000	8 patients, 7 controls	Plasma, PBMCs	PCR	No
Markers of viral infection in monozygotic twins discordant for chronic fatigue syndrome	Koelle et al.	2002	11 patients, 11 controls	Plasma, PBMCs, serum	ELISA, PCR	No
Multiple co-infections (mycoplasma, chlamydia, human herpesvirus-6) in blood of chronic fatigue syndrome patients: association with signs and symptoms	Nicolson et al.	2003	204 patients, 100 controls	Serum	PCR, Southern blot	No
Detection of herpesviruses and parvovirus B19 in gastric and intestinal mucosa of chronic fatigue syndrome patients	Fremont et al.	2009	48 patients, 35 controls	Gastro-intestinal biopsies, serum	qPCR	No
Serological and virological investigation of the role of the herpesviruses EBV, CMV and HHV-6 in post-infective fatigue syndrome	Cameron et al.	2010	20 patients, 10 controls	Serum	qPCR, ELISA	No
No serological evidence for a role of HHV-6 infection in chronic fatigue syndrome	Burbelo et al.	2012	72 patients, 59 controls	Serum	LIPS assay	No
Human endogenous retrovirus-K18 superantigen expression and human herpesvirus-6 and human herpesvirus-7 viral loads in chronic fatigue patients	Oakes et al.	2013	39 patients, 9 controls	Saliva, PBMCs	qPCR	No
Human herpesvirus 6 and 7 are biomarkers for fatigue, which distinguish between physiological fatigue and pathological fatigue	Aoki et al.	2016	97 patients, 113 controls	Saliva	Real-time PCR	No

*CF* chronic fatigue, *CFS* chronic fatigue syndrome, *ELISA* enzyme-linked immunosorbent assay, *FACS* fluorescence-activated cell sorting, *FM* fibromyalgia, *IFA* Immunofluorescence assay, *LIPS* luciferase immunoprecipitation systems, *nPCR* nested polymerase chain reaction, *PBMCs* peripheral blood mononuclear cells, *PCR* polymerase chain reaction, *qPCR* quantitative polymerase chain reaction, *REA* restriction endonuclease analysis, *UCF* unexplained chronic fatigue

Several groups have also reported co-infections of EBV with HHV-6 in ME/CFS patients [63]. Serological analysis has revealed a higher frequency of antibodies to EBV VCA in patients than controls. In addition, these serum antibodies could persist for several years, indicating that immune disturbances may allow viral reactivation [71]. An abnormal immune response to EBV infection has been reported in ME/CFS cases; however, later publication showed no differences of EBV prevalence among patients and controls [72, 73]. Although Manian and colleagues found higher titers of IgG class antibodies against EBV viral capsid antigen in patients than in controls, they did not find a statistically significant difference in titers of antibodies to early antigens of EBV, HSV-1, HSV-2 and HHV-6 in patients compared to healthy controls. Therefore, they did not conclude that there was a significant association between these infections and ME/CFS [74]. Elsewhere, ME/CFS patients’ EBV antibody titers were not significantly different compared to controls [75]. In 1991, Landay et al. found antibodies to EBV early antigen significantly more frequently among ME/CFS patients than healthy adults, but they observed equal rates of EBV VCA and EBNA seropositivity among patients and controls [76]. At the same time, other studies showed no correlation between EBV infection, nor CMV infection, and ME/CFS [77, 78]. A very low percentage of EBV-specific IgG class antibodies in patients and controls has been described [79]. Zhang et al. have shown that EBV seroprevalence (VCA IgG) among ME/CFS patients was similar to that of the general population, but VCA IgM titers, EBNA IgG titers, and EBV-related genes were associated with ME/CFS subtypes [80]. Moreover, in this cohort, most ME/CFS patients demonstrated primary infection/reactivation serostatus, while the serostatus of controls most often corresponded to the late phase of EBV infection.

In some reports, EBV was not detected in blood plasma by PCR [61], and no difference was revealed in the frequency of EBV genomic sequences detected in stomach and duodenum biopsies by qPCR [68]. Likewise, other studies using immunofluorescence, enzyme-linked immunosorbent assay (ELISA), PCR and western blot have shown that EBV is present, though not significantly more often in patients with ME/CFS than in controls [81–84].

Recently published data also support the hypothesis on herpesviruses involvement in ME/CFS development due to expression of antibodies against herpesviruses-encoded deoxyuridine triphosphate nucleotidohydrolases (dUTPases) that activates humoral immune response [85]; anti-EBV and HHV-6 dUTPase antibodies were present in 55.4% and 54.06% of ME/CFS patients in one cohort, respectively. The same study found EBV dUTPase antibodies in 29.09% of patients and 25.83% of controls overall from a separate cohort. Compared to controls, the patients had significantly higher anti-EBV dUTPase antibodies.

Serum antibody and genomic sequence analysis of HSV, VZV, EBV, CMV, HHV-6, HHV-7, HHV-8, JC virus, BK virus, and B19V in 22 monozygotic twin pairs, of which one twin met criteria for ME/CFS and the other twin was healthy, revealed no differences between the group of twins with ME/CFS and the healthy twins [61]. Similarly, Cameron and co-workers did not find significant differences in EBV, HHV-6, nor CMV seropositivity and viral loads between 20 ME/CFS patients and 10 controls using qPCR as well as serological assays for HHV-6 IgG, CMV IgM, and EBV IgG VCA p18, IgG EBNA-1, and IgG EA [83]. Landay et al. also failed to find any serological links between these viruses as well as adenovirus, HTLV I/II, HIV, papovavirus, human spumavirus, rubeola, and coxsackie B4 and the pathogenesis of ME/CFS [76] Elevated IgG titers to CMV were demonstrated in only 6/107 (6%) patients with ME/CFS and only rarely were HSV, coxsackievirus, chlamydia, campylobacter, yersinia or candida infections observed [81]. Likewise, serum analysis revealed no correlation between ME/CFS and CMV, HSV-1, HSV-2 and adenovirus in another report [20]. However, analysis of more ME/CFS patients and controls showed the presence of CMV IgM p52 and CM2 antibodies in 16/34 patients and none of the controls, suggesting an etiologic role of CMV in ME/CFS [86]. HHV-8, which has been infrequently studied in this disease, was found in 2/35 (5.7%) ME/CFS patients and in 1/25 (4%) controls [22].

Although an association of ME/CFS with viral infections has not been demonstrated in all studies, ME/CFS can be triggered by various factors, and infections could contribute to a subgroup of ME/CFS patients [66, 83]. Moreover, autoimmune, immune, metabolic and psychological disturbances could emerge due to infectious disease [2].

## Enteroviruses

Single-stranded positive-sense RNA viruses from the *Enterovirus* genus belong to the *Picornaviridae* family. They comprise a large group of more than 70 different enteroviruses that have the ability to infect humans. Although enteroviral infections in humans are frequently asymptomatic, they can exert a variety of symptoms during acute infections. In addition, chronic enteroviral infections have been implicated in myocarditis [87–89] and in juvenile-onset type-1 diabetes [90].

The role of enterovirus infection as a potential factor in the etiology of ME/CFS has been suspected for about 3 decades. At the same time, it has been largely disputed, as the data from the literature are controversial. RNA of enteroviruses has been found in variety of specimens (blood, stool, gastric and muscle biopsy) from ME/CFS patients more frequently compared to control subjects (Table 2). Clements et al. detected enterovirus-specific sequences in 36/88 (41%) serum samples from chronic fatigue patients, 22/82 (27%) acutely ill individuals, and 3/126 (2.4%) healthy individuals [91]. In further analysis, the same group determined the prevalence of enterovirus DNA and neutralising antibody in ME/CFS patients. Whereas enteroviral sequences in serum were found by PCR more frequently in the ME/CFS group (42%) than in the control group (9%), there were no statistical differences regarding coxsackievirus B antibodies in the neutralisation tests (positive: ME/CFS 34%, controls: 41%) [92].

**Table 2:** Publications on analysis of enteroviruses in myalgic encephalomyelitis/chronic fatigue syndrome

Publication title	Author	Year	Study participants	Sample type	Method	Correlation assumed
Enteroviral RNA sequences detected by polymerase chain reaction in muscle of patients with post-viral fatigue syndrome	Gow et al.	1991	60 CFS, 41 controls	Muscle biopsies	PCR	Yes
Persistence of enterovirus RNA in muscle biopsy samples suggests that some cases of chronic fatigue syndrome result from a previous, inflammatory viral myopathy	Bowles	1993	148 CFS, 152 controls	Muscle biopsies	Hybridization	Yes
Simultaneous measurement of antibodies to Epstein–Barr virus, human herpesvirus 6, herpes simplex virus types 1 and 2, and 14 enteroviruses in chronic fatigue syndrome: is there evidence of activation of a nonspecific polyclonal immune response?	Manian	1994	20 CFS, 20 controls	Serum	Coxsackievirus B1, B4 antibody titer	Yes
Comparison of Coxsackie B neutralisation and enteroviral PCR in chronic fatigue patients	Nairn et al.	1995	100 patients, 100 controls	Serum	PCR, antibody in neutralisation assay	Yes for PCR, not for NA
Detection of enterovirus—specific RNA in serum: the relationship to chronic fatigue	Clements et al.	1995	88 patients, 126 controls	Serum, buffy coat, stool	PCR	Yes
Phylogenetic analysis of short enteroviral sequences from patients with chronic fatigue syndrome	Galbraith	1995	238 CFS, 130 controls	Serum, throat swaps	PCR	Yes
Detection of enterovirus in human skeletal muscle from patients with chronic inflammatory muscle disease or fibromyalgia and healthy subjects	Douche-Aourik et al.	2003	30 CFS/Fibromyalgia patients, 29 controls	Muscle biopsies	RT-PCR	Yes
Enterovirus related metabolic myopathy: a postviral fatigue syndrome.	Lane et al.	2003	48 CFS, 29 controls	Muscle biopsies	RT-PCR	Yes
Chronic fatigue syndrome is associated with chronic enterovirus infection of the stomach	Chia et al.	2008	165 CFS,34 controls	Gastric biopsies	VP-1 staining, RT-PCR	Yes
Acute enterovirus infection followed by myalgic encephalomyelitis/chronic fatigue syndrome (ME/CFS) and viral persistence	Chia et al.	2009	3 CFS	Gastric biopsies, blood	VP-1 staining, RT-PCR	Yes
Chronic fatigue syndrome: clinical condition associated with immune activation	Landay et al.	1991	63 CFS, 40 controls	Serum	Coxsackievirus B4 antibodies	Prevalence yes, titer not
Studies on enterovirus in patients with chronic fatigue syndrome	Gow et al.	1994.	131 CFS, 101 controls (neuromuscular disorders)	Muscle biopsies	PCR	No
No findings of enteroviruses in Swedish patients with chronic fatigue syndrome	Lindh et al.	1996	29 CFS	Muscle biopsies	PCR	No
Investigation by polymerase chain reaction of enteroviral infection in patients with chronic fatigue syndrome	McArdle	1996	34 CFS	Muscle biopsies	PCR	No
Enteroviruses and the chronic fatigue syndrome	Swanink	1994	76 CFS, 76 controls	Stool, serum	PCR, antibodies	No
Viral serologies in patients with chronic fatigue and chronic fatigue syndrome	Buchwald	1996	508 CFS, 30 controls	Serum	Coxsackievirus B antibodies	No

*CFS* chronic fatigue syndrome, *NA* neutralising antibody, *PCR* polymerase chain reaction, *RT-PCR* reverse transcription polymerase chain reaction

Gow et al. reported on the detection of enteroviral RNA in muscle biopsies from 53% of 60 ME/CFS in comparison to 15% of 41 controls [93]. Furthermore, quadriceps muscle biopsy samples from 20.8% of the 48 ME/CFS patients were found to be positive for enterovirus sequences by reverse transcription (RT)-PCR, while all 29 control samples were negative [94]. In a study by Bowles et al. [95], molecular hybridization detected enteroviral RNA in muscle biopsies from 41 of 148 patients with ME/CFS, 25 of 96 patients with inflammatory muscle disease, and only two of 152 controls. In an earlier study, the same group had postulated that the enteroviral persistence in the muscle might be caused by a defect in the control of viral RNA synthesis as they found approximately equal amounts of enteroviral positive and negative RNA strands, in contrast to lytic infections, in which positive RNA strands dominate [96]. They speculated that persistence of viral RNA without synthesis of viruses could contribute to muscle dysfunction. In 2003, detection of enteroviral RNA without production of viral proteins was reported by Douche-Aourik et al., whose analysis of muscle biopsy samples revealed virus RNA positive samples in 13% (4/30) of ME/CFS patients and none of the controls [97]. Enteroviral VP-1 protein could not be stained by immunohistochemistry in any of the samples.

In 2008, Chia et al. found that 135/165 (82%) antrum biopsy specimens stained positive for enterovirus VP1 within parietal cells, whereas significantly fewer controls were stained positive (7/34, 20%) [98]. Enterovirus RNA was detected in 9/24 (37%) paraffin embedded biopsy samples, and only 1/21 controls had detectable enterovirus RNA. One out of 3 patients had detectable enterovirus RNA from two follow-up samples taken 4 years apart, whereas 5 patient samples showed transient growth of non-cytopathic enteroviruses [98]. In another study from the same group, 3 patients with acute enteroviral infection developed symptoms of ME/CFS during follow-up. Enteroviral persistence was demonstrated by detection of enteroviral RNA sequences in gastric biopsy specimens and in peripheral blood leukocytes (PBL) by qualitative RT-PCR [99]. It was also shown by Galbraith et al. that enteroviruses, 19/20 of which were substantially different from previously described enteroviruses, can establish persistent infection, and in some cases, they can lead to the manifestation of ME/CFS [100]. In the group of patients who had been referred for assessment of fatigue, 44/238 serum samples and 29/175 throat swab samples were positive by enteroviral PCR assay, while sera from 3/130 healthy individuals were positive using the enteroviral PCR assay [100]. In a following study, eight individuals with ME/CFS were positive for enteroviral sequences, which were detected by PCR in two serum samples taken at least 5 months apart [101]. In addition, serological evidence has indicated that enteroviral activity may contribute to ME/CFS; increased titers of antibodies against coxsackieviruses B1 and B4 have been found among patients [74], and in a separate group, enterovirus IgG class antibodies were present in 49% of ME/CFS patients. Within this cohort, acute infections were observed in 5% of patients [80]. The results of antiviral treatment provided additional arguments in support of a potential role for enteroviruses in the pathogenesis of ME/CFS, as 7 out of 10 ME/CFS patients with persistent enterovirus infection were successfully treated with alpha interferon and ribavirin or a combination of alpha and gamma interferon [102].

A study focussing on the analysis of immune activation in ME/CFS found a significantly higher prevalence of coxsackievirus infection in 63 ME/CFS patients (90%) in comparison to 40 controls (65%). However, geometric mean antibody IgG titers were similar in both groups [76]. In contrast, another study found elevated IgG-titers against coxsackievirus in only 6% of 107 ME/CFS patients, arguing against a major role of active coxsackievirus infection in ME/CFS, at least in that cohort.

Other studies were not able to detect enterovirus infection in ME/CFS patients’ blood, stool, serum, cerebrospinal fluid (CSF) and muscle biopsy samples [76, 81, 103–105]. In addition, there were no significant differences in the rates of detection of enteroviral RNA in muscle biopsies from a group of patients with ME/CFS (26.4%), compared with a group of patients with other neuromuscular disorders (19.8%). It was concluded that persistent enterovirus infection is unlikely to play a pathogenic role in ME/CFS, although an effect in initiating the disease process cannot be excluded [106].

## Human parvovirus B19

B19V is an immunomodulating single-stranded DNA virus belonging to the *Parvoviridae* family, the *Parvovirinae* subfamily, and the *Erythrovirus* genus. It was discovered in 1975 in the serum of an apparently healthy donor [107].

B19V was first associated with a human disease in 1981 [108]. It is frequently detected in children, and consequently, 60–80% of adults have antibodies against this virus. It can cause rash, *Erythema infectiosum* or the fifth disease, arthralgia, various skin lesions, neutropenia, liver and lung disorders, papular-purpuric gloves and socks syndrome, hepato-biliary diseases, cardiac syndromes, autoimmune and neurological diseases, transient aplastic crisis with a short life-span and aplasia of red blood cells that is observed in immunocompromised patients [109, 110]. Viral DNA is eliminated from the serum within 4–5 months, and antibodies rapidly decrease. Many years after the primary infection and acute phase, B19V can persist in an organism, and persistent B19V infection has been investigated as a possible etiological agent in cases of encephalitis, encephalopathy, arthritis, autoimmune processes, fatigue and ME/CFS [111, 112].

Initial studies using PCR to examine the presence of B19V DNA in serum and bone marrow aspirates did not find evidence of involvement of B19V infection in bone marrow dysfunction in any of seven ME/CFS patients. Out of these 7 patients, only 1 patient had B19V specific antibodies [113]. Additionally, an analysis of 22 monozygotic twins revealed the presence of B19V genomic sequences in only one of the healthy twins as detected by PCR [61].

A link between B19V infection and subsequent onset of ME/CFS (1–3 years post-infection) has been published [114]. The results from this analysis revealed a significant association between the development of ME/CFS and high stress levels, as determined through the use of questionnaires, during acute B19V infection. Later, B19V DNA was found in plasma samples from 3/58 ME/CFS patients and 2/49 healthy controls, while IgG class antibodies were observed in 52% and 57% of patients and controls, respectively, and IgM class antibodies not present at all [115]. Due to the detection of B19V DNA in gastro-intestinal biopsies from 40% of patients and fewer than 15% of controls, some researchers have concluded that, at least in a subset of patients, B19V could be involved in the pathogenesis of ME/CFS [68].

While analysing the presence of B19V infection markers in the serum of 200 ME/CFS patients and 200 healthy blood donors using real-time PCR, B19V DNA was detected in 11 patients with ME/CFS but in none of the healthy blood donors. A significant difference was not revealed in B19V seroprevalence (the proportion of individuals with anti-B19 VP2 IgG class antibodies) between patients with ME/CFS, 75% of whom were seropositive, and donors, who had a total seroprevalence of 78%. Meanwhile, anti-B19V VP2 IgM class antibodies were found in 4 patients. In addition, 41.5% of patients and only 7% of donors had IgG class antibodies against non-structural (NS1) protein, whereas B19V specific NS1 IgM class antibodies were found in 3 patients and one donor. The presence of B19V specific NS1 antibodies indicates a severe and persistent or chronic B19V infection; in this study, 73% of those with anti-NS1 antibodies suffered from joint pain, and positivity for these antibodies in ME/CFS patients was associated with greater expression of the human ME/CFS-associated genes NHLH1 and GABPA [116]. Another report described finding anti-VP2 IgG class antibodies in 74% of the analysed patients with ME/CFS and IgM in 1 patient. As was the case in the aforementioned study by Kerr et al., B19V seroprevalence among ME/CFS patients was shown to be similar to the percentage in the general population [80]. More recently, B19V VP2 specific antibodies were detected in plasma samples from 85% of the ME/CFS patients and 61% of practically healthy individuals [62]. Moreover, active B19V infection was detected in 28% of ME/CFS patients compared to 2% of controls using nested PCR, and active infection was linked to an increased frequency of joint pain [62]. In spite of these studies, there is no consensus on B19V as a causative agent of ME/CFS.

## Retroviruses

Xenotropic murine leukemia related virus (XMRV) belongs to the *Retroviridae* family, *Orthoretrovirinae* subfamily and *Gammaretrovirus* genus, which was identified in 2006 while studying the lack of ribonuclease L coding antiviral gene RNASEL function in patients with prostate cancer [117]. The genome has 95% homology with several endogenous murine retroviruses and 94% homology with exogenous murine retroviruses [10]. However, Paprotka et al. concluded that XMRV is probably a recombinant virus, which was generated by the recombination of two murine retroviruses (pre-XMRV1 and pre-XMRV-2) during passaging of prostate cancer cell lines in nude mice within a time period ranging from 1993 to 1996 [118].

XMRV was discovered in patients with prostate cancer, and publications reported potential associations between the virus and other diseases. The observations of RNase L proteolysis in PBMC from patients with ME/CFS and infectious-like chronic immune system activation led to examination of XMRV in these patients [119, 120]. The XMRV *gag* gene sequence was detected by nested PCR in PBMCs of 67% out of 100 patients PBMCs but only in 3.7% out of 218 healthy donors PBMCs, and further analysis revealed 99% sequence identity with XMRV by sequencing viral genomes from 3 patients [120]. However, *Science* retracted this article in 2011, citing poor quality control in the experiments, the omission of important information regarding treatment of the ME/CFS-patient PBMCs, and scepticism of the validity of the study [121].

In 2010, Lo et al. reported on the detection of MLV-related virus *gag* gene sequences in 86.5% out of 37 patients and 6.8% out of 44 control group individuals [122]. However, in 2012, the authors retracted the publication. Later, many researchers published their efforts to detect XMRV in patients with ME/CFS and donors using serological and molecular methods. No evidence linking XMRV to ME/CFS was found in studies carried out in Germany, China, Sweden, the United Kingdom, Japan, the United States of America, Canada, the Netherlands, Latvia and Italy [123–135]. The presence of XMRV or MLV-related sequences, antibodies, or infectious virus was not confirmed in large ME/CFS patient groups, including a subset of the patients previously reported to be XMRV-positive by Lombardi and colleagues [136].

It has been suggested that the earlier positive findings were based on laboratory contamination [136–140]. Studies have shown that commercial reagents and clinical samples could be contaminated with MLV-related virus genomic sequences containing murine DNA, and cloned or amplified XMRV DNA might be the source of contamination. It could also originate from frequently used XMRV-infected prostate cancer cell line 22Rv1 [141]. Various geographic localizations may explain differences in some results, but not in the same country. Another reason for problems with XMRV detection was thought to be XMRV sequence variation or the presence of XMRV-like viruses. XMRV strain identity is 99%; therefore, the existence of distinct or related viruses is possible, and detection of them with PCR or some other methods can be difficult [10]. After several years of studies, large effort and expenses from clinicians, scientists and patients, it was concluded that there is no association between XMRV and human diseases, and positive results were consequences of contamination [142].

Studies on several other retroviruses (HTLV-I and -II, HIV-1/2, spuma viruses) in ME/CFS were performed. Whereas DeFreitas reported on the presence of antibodies to HTLV-II and retroviral sequences in blood cells in patients with ME/CFS, these findings could not be reproduced by other groups [76, 143, 144]. Therefore, the theory of retroviral etiology in ME/CFS is not currently supported.

## Ross River virus

Another post-viral fatigue-causing virus is a single-stranded positive-sense RNA virus—Ross River virus (RRV), which belongs to the family *Togaviridae*, genus *Alphavirus*. Mosquitoes transmit this viral infection from infected animals to humans, and symptoms of the infection overlap with the symptoms of ME/CFS. Back in 1996, Selden and Cameron published a study in which such symptoms as joint pain, persistent tiredness, lethargy, myalgia, lymphadenopathy, headache, and depression were observed even 30 months after infection with RRV in South Australia [145].

Using an antibody-dependent enhancement mechanism, RRV has been found to infect macrophages and enable suppression of specific antiviral genes expressed by these cells, which results in unimpeded replication [146]. An additional study by these two authors showed suppression of functional activity of STAT1 and NF-κB transcription factor protein complexes due to antibody-dependent enhancement of RRV infection [147].

Later, acute RRV or EBV infection followed by fever, malaise, pain, fatigue, and mood and concentration disorders were correlated with elevated levels of pro-inflammatory cytokines [148]. Analysing the same patient cohort in Australia, Hickie and colleagues studied the prevalence and course of post-infectious fatigue and chronic fatigue following acute RRV, EBV and *Coxiella burnetii* infection, demonstrating that severity of acute viral infection and the host response to it may determine the course of post-infectious syndrome and ME/CFS [82]. More recent attempts to find a possible illness-specific signature of peripheral blood gene expression in patients with post-infectious fatigue caused by RRV, EBV, or *Coxiella burnetii*, was not successful [149]. Subsequently, this team hypothesized that inflammatory cytokines influence the CNS, resulting in neurocognitive disturbances following acute infection, and therefore, certain infectious agent may not determine symptoms. Moreover, genetic predisposition of specific cytokine expression has been found to affect cognitive manifestations, particularly during acute infection [150].

## Molecular mechanisms behind viral pathogenesis in ME/CFS

**Fig. 1:**
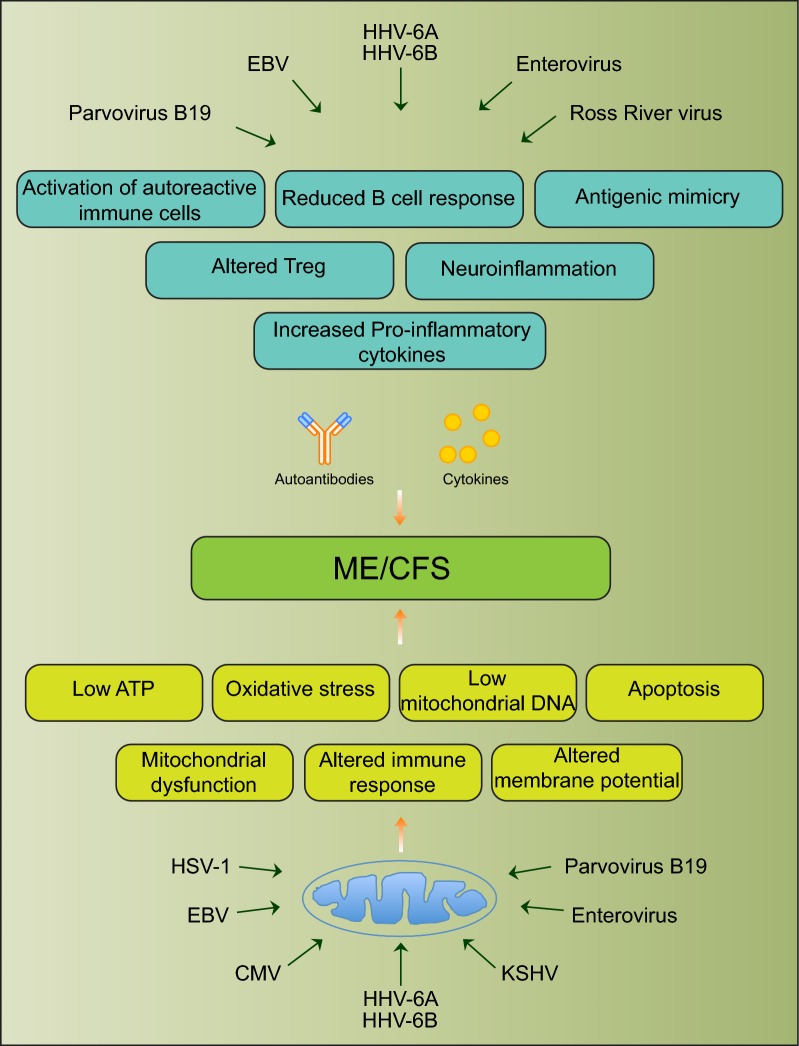
Schematic diagram showing various viral pathogens potentially associated with ME/CFS and possible molecular mechanisms altered by these pathogens that can contribute to ME/CFS development

Viral infection can initiate a multitude of physiological changes in host cells that can contribute to ME/CFS development (Fig. 1). Viral pathogens frequently associated with ME/CFS are also known to alter various molecular processes in host cells that define clinical conditions of ME/CFS (Table 3). Three of the most frequently discussed molecular processes, namely immune cell alterations, mitochondrial modulation and autoimmunity, are described in brief within this section.

**Table 3:** Possible viral contributions towards ME/CFS

Viruses	Contribution in ME/CFS
Human herpesviruses	Persist after primary infection in latent phase and can reactivate causing lytic virus replication Infected cells are recognized by immune system resulting in chronic inflammation that causes ME/CFS symptoms Disturb first class major histocompatibility complex (MHC) molecules presenting virus antigen Alter NK, T and B cell function Modify expression of cellular transcripts Trigger immune dysregulation Alter cytokine production resulting in appearance of ME/CFS typical symptoms Contribute in affecting signalling pathways to proper immune response Activate humoral immune response by herpesviruses-encoded dUTPases Infect neurons and immune cells to impair CNS capillaries and micro-arteries, leading to brain damage Produce a pro-inflammatory environment and autoimmune activity Damage tissue, leads to inflammation and may activate auto-reactive T and B cells, thereby contributing to autoimmunity Local virus-associated inflammation of nervous structures results in altered CNS and PNS signalling Alter ATP homeostasis, increase ROS, change mitochondrial metabolism and modulate mitochondrial DNA content
Enteroviruses	Infect various tissue (blood, gastric, muscle, brain) and stool Persistence of viral RNA could contribute to muscle dysfunction Induce tissue damage Dysregulate host microRNAs Induce greater oxidative stress, inflammation, and pro-inflammatory M1 macrophage activity Induced inflammation can result in bystander activation of auto-reactive cells Coxsackie B4 virus infects beta cells leading to NK cell induced non-destructive inflammation Severe acute enterovirus 71 infection diminishes number of NK and T cells and induces ROS accumulation
Parvovirus B19	After primary infection and acute phase can establish persistent infection and lead to the manifestation of ME/CFS Interacts directly with cells leading to a more aggressive fibroblast function and degradation of cartilage matrix Active infection is linked to an increased frequency of joint pain VP1 protein affects arachidonic acid metabolism promoting inflammatory reactions NS1 protein stimulates pro-inflammatory cytokines production causing local inflammation Activates NK cells Causes neuroinflammation Contributes to greater expression of the human CFS-associated genes NHLH1 and GABPA May induce autoimmunity
Retroviruses	No contribution
Ross-River virus	Infect macrophages using antibody-dependent enhancement mechanism Suppresses transcription and translation of antiviral genes Generate neurocognitive manifestations affected by functional polymorphisms in cytokine genes Cause joint pain, persistent tiredness, lethargy, myalgia, lymphadenopathy, headache and depression

### Virus-induced alterations to immune cells

One commonality among the viruses linked to ME/CFS appears to be the ability to establish persistent infections. In order to do that, viruses must bypass and evade immune cells, and in doing so may alter immune cell functions. While several viruses may contribute to ME/CFS, the immunomodulatory capacity of the viral species, perhaps paired with its ability to establish persistent infection, may underlie its pathological potential in the setting of ME/CFS. Elevated activity of antiviral enzyme 2′-5′-oligoadenylate synthetase (OAS) in PBMC from patients with ME/CFS has been observed. This protein is induced by IFN-α and IFN-β, and degrades viral RNA and inhibits virus replication. Therefore, it plays an important role in the response against viral infections. The level of OAS correlates with severity of ME/CFS, suggesting that a chronic virus infection could be the cause of ME/CFS [151]. Moreover, proof of humoral immune response activation by herpesviruses-encoded dUTPases in patients with ME/CFS has been published [85].

The establishment of a persistent infection is influenced by immunosuppression and activated immune complexes, which may cause chronic inflammation [15, 152, 153]. Chronic immune system activation is accompanied by alterations in regulation of cytokine production [154], and stimulated lymphoid cells express or induce the expression of various cytokines in other cells that can set the stage for pathological manifestations [155]. Low-level inflammation and activation of cell-mediated immunity is observed in ME/CFS cases and the high level of TNF-α correlates with several clinical symptoms; therefore, an increase of inflammatory mediators might explain symptoms of the disease [156]. Likewise, it is possible that a viral infection causes dysfunction in cellular immunity, which consequently induces viral reactivation. Subsequently, viral proteins facilitate cytokine secretion, resulting in appearance of typical ME/CFS symptoms, such as fatigue, fever, sleep and cognitive disorders [7]. Chronic pain may be caused by inflammatory signals that are spread by glial cells activated by inflammatory cytokines and neuronal stimulation [153, 157].

In establishing persistence, viruses may induce immune disturbances directly and indirectly; for instance, they may infect cells involved in mediation of cellular and humoral immune response, and through indirect interactions, they might alter cell surface receptor expression as well as cytokine and chemokine expression levels, leading to local inflammation [158]. Infection of cells by enteroviruses affects cellular miRNA expression, which may result in dysregulation of immune pathways and cytokine production, and these viruses can reduce expression of type I and III interferon, which are primarily produced by natural killer (NK) and T cells [159–161]. Herpesviruses like HHV-6 are also able to alter expression of cellular miRNAs in various cell types including NK cells [162, 163], as well as cellular expression of NK cell receptors [164], and they may express their own miRNAs that aid in immune evasion [165]. Downregulated cytotoxic activity of NK cells in ME/CFS could be linked to a decreased expression level of NK cell activating receptor NKG2D [166], the ligand of which is down-regulated by HHV-6B [164]. In addition, the expression of viral homologs of cytokines/chemokines and cytokine/chemokine receptors by herpesviruses can impact immune pathways [167, 168]. Other herpesvirus-induced alterations to ligand/receptor signalling between NK and associated cells could also contribute to the pathogenic mechanism of the viruses in ME/CFS are described [169].

Enteroviruses, as well as most of the herpesviruses, use mechanisms that disturb first class major histocompatibility complex (MHC) molecules presenting virus antigen [167, 170, 171]. The HHV-7 U21 gene product interrupts viral antigen presentation to cytotoxic T cells that causes MHC class I molecules degradation in lysosomes. Besides interfering with the host cytotoxic T cell response, U21 lessens NK cell cytotoxicity [172]. Such changes of immunological parameters as a decreased count of CD3^+^ and CD4^+^ T cells, an increase of CD95^+^ and a decrease of CD4^+^/CD8^+^ ratio are observed in patients with a concurrent active HHV-6 and HHV-7 infection [64]. Other studies also show CD4^+^ T cell response to HHV-6 [173].

In vitro studies show that a possible mechanism of action for B19V is direct virus interaction with cells leading to more aggressive fibroblast functionality and degradation of cartilage matrix. Moreover, the activity of capsid protein VP1 affects arachidonic acid metabolism promoting inflammatory reactions, and the B19V non-structural NS1 protein also stimulates pro-inflammatory cytokine production, causing local inflammation [174] that might account for such ME/CFS clinical manifestations as fatigue, lymphadenopathy, joint pain, and muscle pain [68, 175, 176]. Enteroviruses [177, 178] and herpesviruses may also contribute to these signs and symptoms through up-regulation of pro-inflammatory cytokines [179].

Some viruses, like RRV, are able to infect macrophages using an antibody-dependent enhancement mechanism, suppressing antiviral genes and thereby resulting in replication [146]. The antiviral response to RRV is diminished by suppression of transcription factor protein complex activity [147]. In the case of acute RRV infection, functional polymorphisms in cytokine genes may affect the influence of inflammatory cytokines in the CNS and resulting neurocognitive manifestations [150].

### Viruses and mitochondrial modulation

ME/CFS is considered to be a mitochondrial disease [180]. Some of the characteristic features of ME/CFS involve altered adenosine triphosphate (ATP) homeostasis [181, 182], increased reactive oxygen species (ROS) [183], changed mitochondrial metabolism [184, 185], and modulation of mitochondrial DNA content [182, 186, 187]. Many of the aforementioned viruses modulate host mitochondria in a variety of ways that can potentially present plausible explanations regarding the involvement of these viruses in ME/CFS. Viruses have evolved distinctive strategies to alter mitochondrial metabolism and bioenergetics, which may allow enhanced viral replication and provide anti-viral defence.

HSV-1 infection decreases cellular ATP levels and mitochondrial membrane potential [188], and HSV-1 anti-apoptotic protein gJ has been shown to induce ROS formation [189]. In vitro experiments in mammalian cells have shown rapid and complete degradation of host mitochondrial DNA by HSV-1 [190]. Evidence of mitochondrial dysfunction has been associated with post-infective fatigue after EBV infection [191], and EBV is known to alter mitochondrial dynamics through direct interaction [192]; the immediate early protein of EBV, Zta, interacts with mitochondrial single stranded DNA binding protein, leading to reduced mitochondrial DNA (mtDNA) replication and enhanced viral DNA replication [193]. HHV-6B improves viral infection through direct interaction of its U95 protein with human GRIM-19 protein [194], and the virus increases oxidative stress during persistent infection by reducing glutathione reductase activity [195]. During productive infection, HHV-6A can cause apoptosis through a caspase-dependent pathway accompanied by altered mitochondrial morphology and lower transmembrane potential [163, 196]. Similarly, latent CMV has been associated with greater oxidative damage [197]. HHV-8 modulates mitochondrial antiviral signalling via its interferon regulatory factor 1 [198]. In addition, porcine and canine parvovirus infection induces depolarization of the mitochondrial membrane, damage to the organelle’s structure [199], and ROS accumulation [200, 201]. Enterovirus 71 also induces ROS accumulation [202] for its successful replication.

### Viruses and autoimmune signature

Autoimmune signature in ME/CFS has recently become a subject of intense research [29, 203]. Viral pathogens can contribute to autoimmune diseases in variety of ways. Because the viruses associated with ME/CFS are also commonly detected among healthy individuals, it may be the case that underlying immune dysfunction in the host acts as a predisposing factor in development of the disorder. Viruses may trigger immune dysregulation, but an individual may also be predisposed to either an exceptionally strong acute infection, an inability to completely clear the virus, or both. Subsequently, the abnormal immune profile post-acute infection may allow for continuous reactivation and incomplete clearance of pathogens, resulting in tissue damage and an overactive yet ineffective immune response leading to inflammation and autoimmune changes. The absence of strong viremia indicates that the viruses that are present are likely quite tissue-specific, and data suggests that the viral activity consists of greater “latency-associated replication”, as was noted for EBV [72]. The success of valganciclovir (active against CMV and HHV-6) and valacyclovir (active against HSV, VZV, and EBV) [86, 204] as well as rituximab and immunoadsorption of ß2 autoantibodies in subsets of ME/CFS patients suggests that both the viral activity itself and the immune response against/resulting from viral infection may contribute to the signs and symptoms of the illness [205, 206].

Enteroviruses, particularly B coxsackieviruses, are implicated in type 1 diabetes mellitus, which they may contribute to through dysregulation of host microRNAs [207] and induction of greater oxidative stress, inflammation, and proinflammatory M1 macrophage activity [208]. Chronic enterovirus-associated systemic or local inflammation, as a consequence of a weak innate immune response and virus-induced tissue damage, may result in bystander activation of auto-reactive cells. On the other end of the spectrum, an overactive innate immune response may also result in inflammation during the initial acute infection, leading to the same outcome [209]. This scenario has been proposed for enteroviruses in type 1 diabetes, but the theory may be more broadly applicable to other viruses and autoimmune conditions. Activation of auto-reactive bystander cells has also been proposed as a pathogenic factor in systemic lupus erythematosus [210], as has molecular mimicry.

The viruses discussed herein are capable of activating NK, B, and T cells, modifying expression of their cellular transcripts, altering cytokine production, and affecting signalling pathways integral to the proper functioning of the immune response, potentially producing a pro-inflammatory environment and autoimmune activity. For example, NK cells induce non-destructive inflammation in response to beta cell infection by coxsackie B4 enterovirus [211] and are activated by acute B19V infection [212], but NK and T cells are diminished in number during severe acute infection of enterovirus 71 [213]. HHV-6A-infected T cells express many miRNAs associated with inflammation and autoimmunity [214], and persistent HHV-6A infection is associated with altered NK cell profiles in cases of Hashimoto’s thyroiditis and acute necrotizing encephalopathy. These infections were characterized by incomplete clearance of the virus and a greater abundance of peripheral CD56bright NK cells [215, 216]. This environment of heightened cytokine secretion paired with the ineffectual clearance of infection may activate auto-reactive T and B cells, thereby contributing to autoimmunity. Indeed, the cellular activation of the CD56bright NK cells correlated with autoantibody levels in subjects with Hashimoto’s thyroiditis.

Interactions between the viruses and innate and adaptive immune cells are dynamic and vary temporally, across viral species, and between individual patients. Accordingly, activity of these cells has been found to vary across studies of ME/CFS and even within studies, according to the time-point [217]. Although cytokine expression and immune cell phenotypes have differed [62, 218], dysregulation of immune cell networks occurs in ME/CFS patients, and a pro-inflammatory milieu appears to predominate. Additionally, it is clear that patients’ immune responses against viruses differ from unaffected individuals. While healthy volunteers did not exhibit changes in cellular proliferation after vaccination with influenza strains, for example, ME/CFS patients displayed significantly higher cellular proliferation in response to stimulation compared to baseline [219]. Baseline T cell proliferation was lower than controls, and post-vaccination proliferation was higher in ME/CFS patients compared to controls, although these findings were not statistically significant. Lower proliferative responses were observed among other ME/CFS patients as well [220], and decreased cytotoxicity of NK and T cells [217], as well as increased levels of cytokine producing CD56bright NK cells [221], have been documented, although findings have not been consistently confirmed between studies.

Failure to produce IgG antibodies against EBV EBNA-1 has been noted, as has low levels of antibody secreting cells specific to EBNA-1 and VCA and an impaired B cell memory response to the virus [72]. Responses of EBV-specific B- and T-cells were suppressed, which has been suggested to result from frequently reactivated EBV and lymphocytic exhaustion. In contrast, reduced CMV and HSV specific B-cell responses were not identified. On the other hand, the presence of antibodies against EBV DNA polymerase and EBV-encoded dUTPase has been detected in the serum of a subset of ME/CFS patients but not in controls [84], and the IgG response to EBV EBNA-6 peptides has been heightened in ME/CFS [73]. Sequences of amino acids in the EBNA-6 repeat region were found to be homologous to the human lactoperoxidase precursor (LPO) and thyroid peroxidase precursor (TPO) proteins as well as the two enzymes ornithine transcarbamylase (OTC) and phosphofructokinase-1 (PFK-1), which have metabolic functions. Of these, only LPO peptide IgG levels correlated with EBNA-6 peptide and protein IgG. However, antigenic mimicry producing cross-reactivity among these proteins is considered as a possible factor behind ME/CFS pathogenesis. Analysis of genetic associations has revealed that the EBV protein EBNA-2 and its related human transcription factors are associated with many autoimmune risk loci, including those involved in MS, rheumatoid arthritis, type 1 diabetes, and systemic lupus erythematosus [222]. NF-kB was similarly associated.

Although systemic immune dysfunction has been observed, local virus-associated inflammation of nervous structures resulting in altered CNS and peripheral nervous system signalling has also been hypothesized as a mechanism behind ME/CFS [223, 224], and neuroinflammation has been observed in patients [225]. EBV [226], HSV [227], HHV-6 [228], CMV [229], VZV [230], enterovirus [231], and B19V [232] can all infect the brain. Notably, HHV-6A is able to induce neuroinflammation in the absence of active replication in a mouse model [233]. HHV-6A has also been found to impair myelin repair in vitro and in vivo using mouse with extensive loss of myelin [234].

HHV-6 has been strongly linked to autoimmune diseases like Hasimoto’s thyroiditis [235, 236], connective tissue disorders and MS [237]. Selective reactivation of HHV-6 has also been shown in patients with autoimmune connective tissue diseases [238]. MS patients are frequently detected with high levels of HHV-6A/B-specific IgG and IgM in the serum and CSF [239]. Myelin basic protein (MBP), one of the auto-antigens implicated in MS pathology shares amino acid sequence homology with the U24 protein from HHV-6 [240]. Hence molecular mimicry is considered as one of the potential mechanisms for HHV-6 mediated autoimmune diseases. Clinical cases showing increased glutamic acid decarboxylase (GAD) antibodies and HHV-6 infection has been reported where antiviral therapy improved patient’s clinical condition [241].

HHV-6B has weaker ties to autoimmunity, but it is thought to contribute to common symptoms of severe drug-induced hypersensitivity syndrome/drug reaction with eosinophilia and systemic symptoms (DIHS/DRESS) when it reactivates strongly during the course of the syndrome. The immune response to the viral activity is considered as potentially more destructive than the viral activity itself and is implicated in visceral organ damage [242]. Notably, initial regulatory T cell (Treg) expansion in this illness contrasts with subsequent exhaustion of Treg cells during remission, which appears to set the stage for the autoimmune sequelae that commonly occur afterward [243]. Autoimmune thyroiditis and diabetes mellitus frequently arise post-DIHS/DRESS, as does chronic fatigue. HHV-6 has been detected in Treg cells in a case of likely DIHS/DRESS [244], and HHV-6A [245] and HHV-6B [246] are also able to induce the development of Treg cells. Both upregulated and reduced levels of Tregs have been reported in ME/CFS [217, 221, 247].

The molecular link between B19V infection and autoimmune disorders is not very clear. However, B19V infection has been associated with development of autoimmune antibodies including rheumatoid factor [110, 248, 249], antinuclear antibody, anti-mitochondrial antibody, smooth muscle antibody, and gastric parietal cell antibody [250]. Recently, B19V infection has been shown to increase levels of cytokines like IL-4, IL-10, IL-12, IL-2 and TNFα in the plasma of rheumatoid arthritis patients [251]. B19V induced upregulation of IL-6 has also been noted for its potential role in autoimmunity [252]. Two of the B19V proteins, a proline-rich small protein [253] and the NS1 protein [254], which also function as a transcription regulator have been intensely studied because of their role in viral pathogenesis. These proteins also contribute to host cell immune modulation through their involvement in cell survival pathways [254, 255]. B19V has been implicated in clinical cases of systemic lupus erythematosus and rheumatoid arthritis where various auto-antibodies could be detected in patients. Intravenous immunoglobulin (IVIG) has been reported to reduce symptoms in some patients with persistent B19V infection, but IVIG administration has also resulted in unexpected worsening of symptoms in a ME/CFS patient [256]. This may indicate that, in some cases, the immune response against persistent infection contributes to the development of clinical symptoms more so than infection-induced tissue damage, and IVIG may contribute to increased inflammation. Heightened viral replication was also detected in this case, which was suggested to be a result of antibody-dependent enhancement.

At least in a subset of patients, the mitochondrial dysfunction and elements of autoimmunity that characterize ME/CFS may be linked to viral pathogenesis. Lack of extensive analysis of molecular mechanisms linking viral pathogens to ME/CFS restricts our understanding of this disease. Future studies need to focus on this aspect of ME/CFS research.

## Conclusions

Currently available data on the role of chronic viral infection with ME/CFS is still controversial, showing potential viral involvement for at least a subgroup of ME/CFS patients. Therefore, it is necessary to assess the presence and markers of viral activity at the initial stage of the disease to evaluate possible etiological factors and conduct longitudinal studies in order to assess active viral infection and symptom severity variations over time. Moreover, results should be compared not only between ME/CFS patients and controls, but also with other co-morbidities to assess specificity of suggested biomarkers.

Considering ME/CFS heterogeneity, the use of clinical characteristics and biomarkers to enable definition of the disease subtypes is crucial. In addition, longitudinal and standardized studies determining ME/CFS course and therapy effectiveness with follow-up measurements in dynamics should be accomplished. This will allow prognosis of the disease development and promote development of a specific definition for diagnostics and a treatment plan.

## Future strategies for development of infection biomarkers in ME/CFS

1. Use of quantitative assays rather than qualitative assays to assess the extent of the viral load instead of simple detection of presence or absence. This may facilitate monitoring of a response to treatment; however, diurnal variations and individual response on treatment should be taken into account. Further comprehensive serological testing may help to identify a signature of active infection.
2. Use of additional biological samples together with blood and serum will be useful in determining the localization and distribution of biomarkers, as well as pathogenicity. Using hair follicles, virus integration can be detected. Similarly throat swab and stool samples can be used for detection of enteroviruses.
3. Functional studies to compliment clinical biomarker studies in order to clarify functions and interactions of genes, transcripts, proteins, and immune cells and molecules in cases of ME/CFS. This will facilitate understanding of the disease aetiology as well as development and maintenance pathways, and thereby, potential prevention and treatment strategies. However, this strategy requires definition of ME/CFS subgroups.
4. Use of high throughput methods to gain broader insight into potential biomarkers for infections by obtaining and analysing large-scale data, which will raise the quality and significance of the research.
5. Confirmation of results by validation studies and multi-centre cohort studies to obtain generalizability of the study and promote implementation of credible biomarkers usable worldwide.
